# MicroRNA Expression Aberration as Potential Peripheral Blood Biomarkers for Schizophrenia

**DOI:** 10.1371/journal.pone.0021635

**Published:** 2011-06-29

**Authors:** Chi-Yu Lai, Sung-Liang Yu, Ming H. Hsieh, Chun-Houh Chen, Hsuan-Yu Chen, Chun-Chiang Wen, Yung-Hsiang Huang, Po-Chang Hsiao, Chuhsing Kate Hsiao, Chih-Min Liu, Pan-Chyr Yang, Hai-Gwo Hwu, Wei J. Chen

**Affiliations:** 1 Institute of Epidemiology and Preventive Medicine, College of Public Health, National Taiwan University, Taipei, Taiwan; 2 Center of Genomic Medicine, National Taiwan University, Taipei, Taiwan; 3 Department of Clinical Laboratory Sciences and Medical Biotechnology, College of Medicine, National Taiwan University, Taipei, Taiwan; 4 Department of Psychiatry, College of Medicine and National Taiwan University Hospital, National Taiwan University, Taipei, Taiwan; 5 Institute of Statistical Science, Academia Sinica, Taipei, Taiwan; 6 Department of Internal Medicine, College of Medicine and National Taiwan University Hospital, National Taiwan University, Taipei, Taiwan; 7 Neurobiology and Cognitive Center, National Taiwan University, Taipei, Taiwan; Wayne State University, United States of America

## Abstract

Since brain tissue is not readily accessible, a new focus in search of biomarkers for schizophrenia is blood-based expression profiling of non-protein coding genes such as microRNAs (miRNAs), which regulate gene expression by inhibiting the translation of messenger RNAs. This study aimed to identify potential miRNA signature for schizophrenia by comparing genome-wide miRNA expression profiles in patients with schizophrenia vs. healthy controls. A genome-wide miRNA expression profiling was performed using a Taqman array of 365 human miRNAs in the mononuclear leukocytes of a learning set of 30 cases and 30 controls. The discriminating performance of potential biomarkers was validated in an independent testing set of 60 cases and 30 controls. The expression levels of the miRNA signature were then evaluated for their correlation with the patients' clinical symptoms, neurocognitive performances, and neurophysiological functions. A seven-miRNA signature (hsa-miR-34a, miR-449a, miR-564, miR-432, miR-548d, miR-572 and miR-652) was derived from a supervised classification with internal cross-validation, with an area under the curve (AUC) of receiver operating characteristics of 93%. The putative signature was then validated in the testing set, with an AUC of 85%. Among these miRNAs, miR-34a was differentially expressed between cases and controls in both the learning (*P* = 0.005) and the testing set (*P* = 0.002). These miRNAs were differentially correlated with patients' negative symptoms, neurocognitive performance scores, and event-related potentials. The results indicated that the mononuclear leukocyte-based miRNA profiling is a feasible way to identify biomarkers for schizophrenia, and the seven-miRNA signature warrants further investigation.

## Introduction

Schizophrenia is a common and often disabling mental illness characterized not only by a varied group of clinical symptoms [1] but also wide-ranging deficits in neurocognitive and neurophysiological functions [2]–[4]. Although the underlying pathophysiology of schizophrenia remains not well understood, dysregulations of its susceptibility genes are likely to converge functionally upon illness risk [5], which may manifest as certain endophenotypes, such as abnormalities in sustained attention, executive function, or event-related potentials [6]–[7]. Biomarkers that reflect these dysregulations for schizophrenia have the potential to substantially improve the clinical management of the disorder and even revolutionize its drug development approaches [8]–[9]. Since brain tissue is not readily accessible for investigation, blood-based expression profiling is increasingly being undertaken to search for potential biomarkers for schizophrenia [10]–[13].

The rationale of searching for peripheral blood-based gene expression aberration for schizophrenia is two-folded. First, constitutional variants of susceptibility genes exhibited only limited association with schizophrenia as revealed in systematic meta-analyses of genome-wide association studies [14]. Hence, an alternative way is to look for measures that can reflect the combined effect of genetic predisposition and environmental exposure, such as aberrations of gene expression. Second, the central nervous system (CNS) may exert its influence on the gene expression of peripheral lymphocytes via cytokines, neurotransmitters, or hormones [15]–[16], which may explain the comparable gene expression levels between the peripheral blood and some CNS tissues [11]–[12], as well as the alterations in the dopamine transporter in the lymphocytes of psychotic patients [17]. Furthermore, the peripheral expression levels of some dysregulated genes were also found to be correlated with certain clinical symptoms and neurocognitive functions in patients with schizophrenia [18].

However, most of previous blood-based gene expression studies on schizophrenia [10]–[13] were limited to the expression of protein-coding genes, in which an enormous number of messenger RNAs (mRNAs) have to be examined using microarray platforms. This approach usually led to a large set of putative mRNAs related to schizophrenia and most of them were not replicated in the other studies [19]. Growing attention has been focused on the regulatory roles of microRNAs (miRNAs), which regulate gene expression by inhibiting the translation of mRNAs [20], as potential biomarkers for schizophrenia [21]–[25]. Since each miRNA can regulate the expressions of hundred of target genes, the number of discriminating miRNA markers would be much less than that of mRNAs. Indeed, in some preliminary array-based miRNA profiling studies, the expressions of less than thirty miRNAs were found to be altered in the *post-mortem* brains of schizophrenia patients [26]–[29]. As exemplified by the parallel changes in the levels of certain mRNAs between the CNS and peripheral blood [15]–[17], it is plausible that some expression aberrations of miRNAs might also be detectable in the peripheral mononuclear leukocytes.

In this study, we aimed to identify miRNAs that might be useful as biomarkers for schizophrenia in a two-stage manner. We first examined array-based miRNA profiles in peripheral mononuclear leukocytes from a learning set of schizophrenia patients and healthy controls, and then the discriminating performance of candidate miRNAs was validated in an independent testing set of cases and controls. The expression levels of the miRNA signature were further evaluated for their correlation with the patients' clinical symptoms, neurocognitive performances, and neurophysiological functions. Possible functions and biological mechanisms implicated in the target genes regulated by the miRNAs were then explored using database prediction.

## Results

There were no differences in age or sex between patients with schizophrenia and healthy controls, whereas the patients' education levels were lower than those of the controls in both the learning and testing sets, and the patients' smoking prevalence was higher than controls' in the testing set (**Table S1**). Patients' mean age at onset was 23.7 for the learning set and 24.6 years for the testing set.

**Figure 1:**
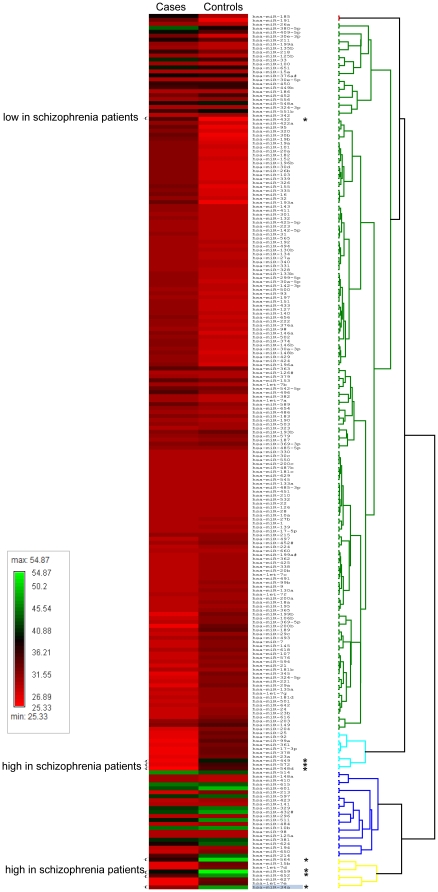
miRNA expression profiling of 221 miRNAs in the learning set of 30 schizophrenia patients and 30 controls. The heatmaps of individual miRNAs for each group of subjects were presented as the averaged rank-sum of the normalized threshold cycle number, with less numbers indicating higher expression levels. The eight differentially expressed miRNAs were marked by an asterisk.

**Figure 2:**
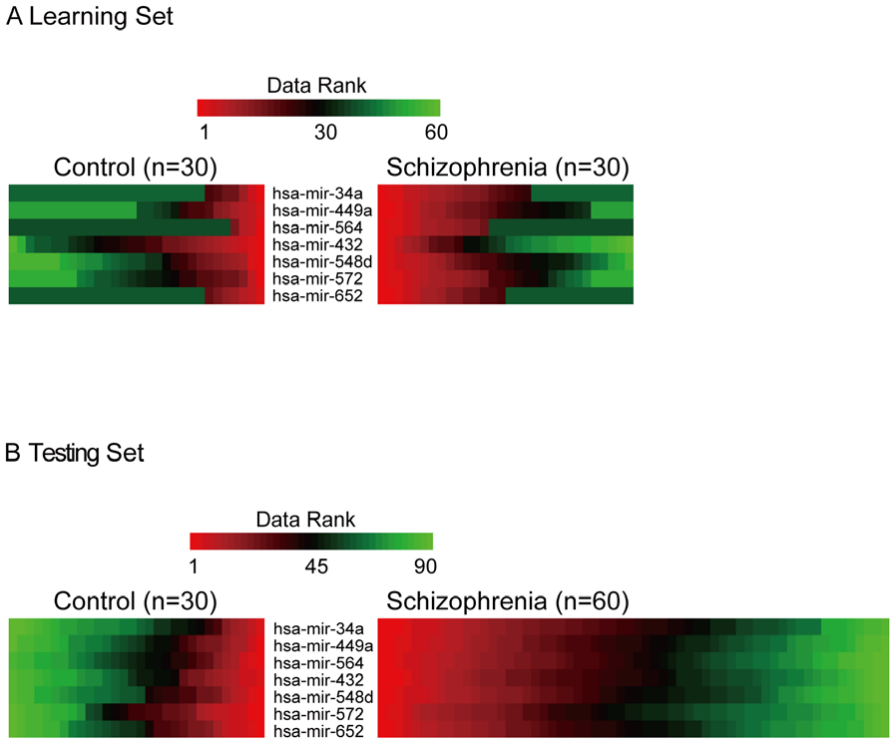
A comparison of: (A) the TLDA-based miRNA expression levels between the schizophrenia patients (*n* = 30) and the controls (*n* = 30) for the seven miRNAs; and (B) quantitative RT-PCR-based miRNA expression levels between the schizophrenia patients (*n* = 60) and the controls (*n* = 30) for the same seven miRNAs. The red, black, and green hues denote relatively high, intermediate, and low expression levels. To emphasize the rank-sum property of the Wilcoxon test, all the expression levels of miRNA were converted to relative expression ranks within each miRNA. Ranks for each miRNA were sorted separately for the control group and disease group before they were placed side-by-side for easier comparison between the two groups and across all putatively informative miRNAs.

Out of the 365 miRNAs assayed using the Taqman Low Density Array (TLDA) in the learning set of 30 schizophrenia patients and 30 controls, the expression levels of 221 (60.5%) were detectable in the peripheral mononuclear leukocytes of ≥30% of the samples in either the schizophrenia patients or the controls. Among them, there were eight miRNAs that were differentially expressed between schizophrenia patients and healthy controls in the learning set based on a criterion of *P*<0.05 in the Wilcoxon rank-sum test (**Figure 1**). From these a seven-miRNA signature (miR-34a, miR-449a, miR-564, miR-432, miR-548d, miR-572 and miR-652) was identified using stepwise logistic regression analysis, with a fold-change ranging from −1.4 to 2.5 (**Table 1**). Among the seven, four in the human database miRNAMap [30] and one in a mouse model [31] were reported to have detectable expressions in the brain, whereas the remaining two did not. The pattern of expression gradients of these seven miRNAs can be distinguished between the two groups, as shown in **Figure 2a**.

**Table 1:** The seven microRNA-signature derived from the learning set (30 patients with schizophrenia and 30 healthy controls).

miRNA name	Chromosomal region	Location	*P* a value	Fold change	Expression levels in the brain
hsa-miR-34a	1p36.23	Intergenic	0.005	2.5	Moderate^b^
hsa-miR-449a	5q11.2	Intron	0.007	1.9	Low^b^
hsa-miR-564	3p21.31	3′ UTR	0.015	2.4	NA
hsa-miR-432	14q32.31	Intergenic	0.022	−1.4	High^b^
hsa-miR-548d	8q24.13	Intron	0.036	1.4	NA
hsa-miR-572	4p15.33	Intergenic	0.038	2.1	Moderate^b^
hsa-miR-652	Xq22.3	Intron	0.049	2.4	Moderate^c^

a based on the Wilcoxon rank-sum test.

b http://mirnamap.mbc.nctu.edu.tw/.

c miR-652 was expressed in the brain and spinal cord during embryonic stages followed by a gradual decrease after birth in a mouse model.

**Figure 3:**
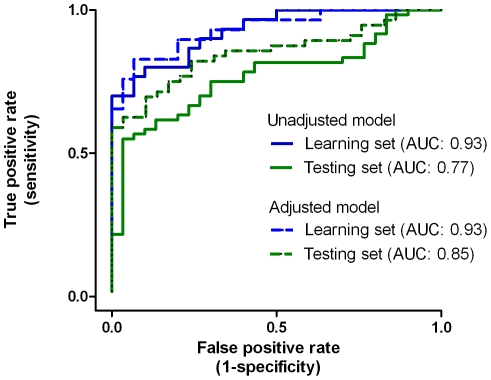
The area under the receiver operative characteristics curve of the seven-miRNA signature in the learning (in blue color) and testing (in green color) sets, respectively, without (in solid line) or with (in dotted line) adjustment for age, gender, education, and tobacco smoking.

As an initial check for the utility of the 7-miRNA signature, the prediction accuracy at an arbitrary cut-off point in the learning set was evaluated using two different methods. For the logistic regression analysis with leave-one-out cross-validation, the raw accuracy rate was 80%, and the adjusted accuracy rate, i.e., with adjustment for potential confounders such as age, gender, education and tobacco smoking, was elevated to 83.1%. When the Support Vector Machine (SVM) [32] was used for the evaluation, the raw and adjusted accuracy rate was 84.3% and 82.1%, respectively. Meanwhile, the global performance of the seven-miRNA signature in distinguishing the schizophrenia patients from the normal controls, the area under the curve (AUC) of receiver operative characteristics, was estimated to be 0.93 for the learning set, regardless of the adjustment for confounders (**Figure 3**). When the disease status was permuted for the seven-miRNA signature, none of the permuted AUCs exceeded the observed value of 0.93, i.e., a significance level of <0.0001 regardless of the unadjusted or adjusted model (data not shown). When the seven miRNAs were randomly selected from the pool of 221 miRNAs for 100,000 times, the mean of these AUCs were 0.69 (SD = 0.06) for the model without adjustment and 0.737 (SD = 0.05) for the model with adjustment for confounders. None of the unadjusted model (*P*<0.00001) and one of the adjusted model (*P* = 0.00001) had an AUC greater than the observed. The histograms of the permuted AUCs for these two models are displayed in **Figure S1**. When the number of miRNAs selected ranged from 3 to 9, the mean AUC increased slightly but remained less than 0.78 (see **Table S2** for the summary of the permutations).

The discriminating accuracy of seven-miRNA signature was then evaluated in an independent testing set of 60 schizophrenia patients and 30 controls using quantitative reverse transcription-polymerase chain reaction (qRT-PCR). The differential expression gradients of the 7-miRNA signature in the testing set is illustrated in **Figure 2b**. The raw and adjusted accuracy rate for the logistic regression analysis with leave-one-out cross-validation was 73.3% and 80.0%, respectively, and the corresponding figure for the SVM was 76.2% and 78.3%, respectively. In terms of the global performance, the AUC was estimated to be 0.77 (without adjustment) and 0.85 (with adjustment), respectively (**Figure 3**). The permutation-based significance level for the testing set was of <0.0001, regardless of the unadjusted or adjusted model.

**Figure 4:**
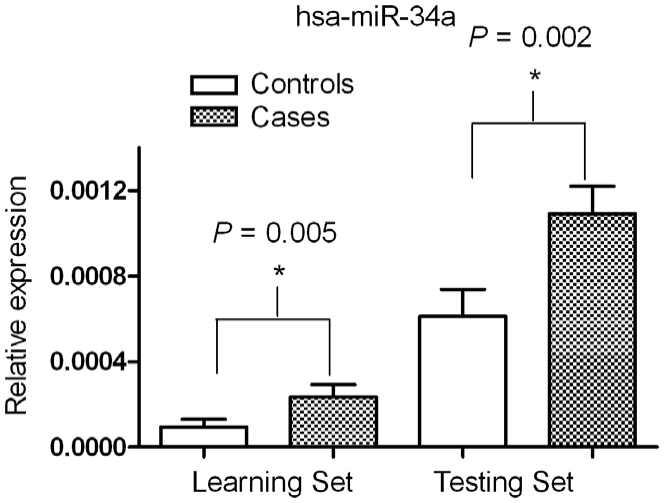
The relations in has-miR-34a miRNA expression levels between two platforms of miRNA quantification, the array-based TLDA vs. individual quantification using quantitative RT-PCR. The differential expressions in hsa-miR-34a between the cases and controls in both the learning set (*P* = 0.005) and the testing set (*P* = 0.002).

Regarding the individual miRNA expression level of the seven miRNAs, the differential expression in hsa-miR-34a between the cases and controls reached statistical significance in both the learning set (*P* = 0.005) and the testing set (*P* = 0.002) (**Figure 4**).

The distributions of subjects' clinical symptoms rated in the Positive and Negative Symptom Scale (PANSS) [33], neurocognitive performances using the Continuous Performance Test (CPT) [34]–[35] and the Wisconsin Card Sorting Test (WCST) [36], and neurophysiological functions measured as P50 suppression in auditory event-related potentials (ERPs) [37] and mismatched negativity (MMN) [38] are shown in **Table S3**. For the pooled sample of schizophrenia patients, some scores on the PANSS, CPT, WCST, and MMN were correlated with the expression levels of certain miRNAs, whereas none of the P50 suppression indexes showed such a correlation (**Table 2**).

**Table 2:** The Spearman correlation coefficients between the seven miRNA signature^a^ and the clinical symptoms of schizophrenia patients from both the learning and testing sets. (Only those with *P*<0.05 are shown here.)

	1	2	3	4	5	6	7
Variable	hsa-miR-34a	hsa-miR-449a	hsa-miR-564	hsa-miR-432	hsa-miR-548d	hsa-miR-572	hsa-miR-652
PANSS (n = 83)							
Negative scale		0.25					
Continuous Performance Test (n = 78)							
Undegraded d′		−0.25	−0.27				
Undegraded false alarm rate		0.23	0.29*				
Degraded ln ·				0.24	0.28*		
Degraded false alarm rate				−0.31*			
Wisconsin Card Sorting Test (n = 78)							
Total errors		0.28*					
Perseverative responses		0.22					
Perseverative errors		0.21					
Non-perseverative errors	0.29*						
Categories achieved		−0.31**				0.24*	
Conceptual level response		−0.29*					
Mismatch Negativity (n = 70)							
Cz						−0.31	
FCz						−0.28	

a The −ΔCt value of each miRNA was standardized (mean = 0, SD = 1) in the learning and testing sets, respectively.

b Scores on the PANSS, MMN, and P50 were adjusted for age, gender, and educational level.

*P<0.01;

**P<0.006.

For the analyses of the predicted miRNA-target genes, 621 genes for three miRNAs (hsa-miR-34a, hsa-miR-449a, and has-miR-432) were obtained using MAMI [39] and 684 genes for two miRNAs (hsa-miR-34a and hsa-miR-449a) were obtained using TargetCombo [40] (see **Table S4** for the setting in each method and the results for each miRNA). Since more information was available for the predicted target genes using MAMI, we further examined whether there were common target genes among the three miRNAs. The results revealed that there were 128 miRNA-target genes shared between hsa-miR-34a and hsa-miR-449a, 1 target gene shared between hsa-miR-34a and hsa-miR-432, and 1 target gene shared between hsa-miR-432 and hsa-miR-449a (see **Table S5** for the complete list).

For each set of predicted target genes, their functions and putative pathways were explored using the software Ingenuity Pathways Analysis (IPA), version 8 (Ingenuity® Systems), with 619 (99.7%) of MAMI-based target genes and 520 (84%) of TargetCombo-based target genes were successfully mapped. The most significantly associated physiological function revealed by the IPA analysis was nervous system development and function for both sets of predicted genes (*P* of Fisher's exact test = 0.000002 for the MAMI and 0.0000013 for the TargetCombo). Since the MAMI-based set covered more miRNAs and predicted target genes, we further explored its canonical biological pathways, with the most significant pathways being cyclin dependent kinase 5 (Cdk5), Notch signaling, and dopamine receptor signaling pathways (**Figure S2**). After correction for multiple testing using the false discovery rate method, both Cdk5 and Notch signaling pathways remained significantly associated with these genes (p<0.001).

## Discussion

Our two-staged search for miRNAs with aberrant levels in blood mononuclear leukocytes revealed a combination of seven miRNA expression alterations to have robustly high discriminating accuracy for schizophrenia. The differential correlation of these miRNAs with patients' negative symptoms, neurocognitive performance scores, and event-related potentials further imply that the aberration of certain functions in the brain might result in alteration of molecular abnormalities in peripheral lymphocytes [15]–[16], [41]. These changes in the post-transcriptional regulatory environment of lymphocytes in patients with schizophrenia not only have implications for the pathophysiology of the disorder [21], [28] but also provide as empirical source of non-invasive biomarkers for schizophrenia.

The use of seven miRNAs as predictors for the disease status has in general more stable prediction accuracy than using a single predictor [42]. Using hsa-miR-34a as an example, which was the most differentially expressed miRNA in the mononuclear leukocytes of patients in both the learning and the testing set, its sole prediction accuracy was only around 50% to 60% (data not shown). Since schizophrenia is a complex neurodevelopmental disorder with a varied group of clinical symptoms, the combined alterations of miRNA expression in the lymphocytes might result from the stresses from different brain regions [15], [41], [43]. This is further supported by the differential correlations of these miRNAs with patients' clinical symptoms and neurocognitive or neurophysiologic measures.

Among the seven miRNAs, the expression level of hsa-miR-34a was found to be altered in the prefrontal cortex of patients with schizophrenia [44]. The expression levels of miR-34a on the hippocampus of rats were also found to be altered in response to mood stabilizers [45]. In cultured cell lines, mood stabilizers could both lower the levels of miR-34a and elevate the levels of GRM7, a confirmed target gene regulated by miR-34a as well as a candidate gene for schizophrenia identified in recent association studies [46]. It should be noted that miR-34a was also significantly up-regulated in the blood mononuclear cells of patients with Alzheimer's disease [47]. Thus, the role of miR-34a over-expression in the pathophysiology of schizophrenia warrants further investigation. Three other miRNAs have also been implicated in neuropsychiatric disorders or neurodevelopment, e.g., miR-432 with autism in human cerebella cortex [48], miR-449 with Alzheimer's disease in cerebrospinal fluid [49], and miR-652 with embryogenesis in the mouse brain and spinal cord [31].

Despite the fact that we do not know whether the aberrant expression of the seven miRNAs were also present in the patients' CNS, the majority of them are detectable in the brain according to the literature [30]–[31]. In addition, some of the seven miRNAs identified in this study were correlated with negative symptoms, neurocognitive dysfunction, and MMN in ERPs in patients with schizophrenia. It is worthwhile to note that miR-449a significantly correlated with most features of the WCST, implying that miR-449a might be sensitive to the executive function activity in the brain. It is plausible that the CNS may exert its influence on the gene expression of peripheral lymphocytes via cytokines, neurotransmitters, or hormones [15]–[16]. Thus, it warrants further investigation regarding which molecules are involved in such influence on peripheral lymphocytes.

Taken together, the aberrant expression of the miRNAs in the peripheral lymphocytes or monocytes of schizophrenia patients could, to some extent, reflect the corresponding dysfunction in the brain, which is compatible with the predicted canonical pathways for these miRNAs, including Cdk5, Notch signaling, and dopamine receptor signaling pathways. Although our prediction analyses of miRNA-target genes and pathways were based on in-silico simulation, some of the miRNA-target genes have been validated in previous studies [50]. Hence, our results are compatible with the implication of the dysregulation of these genes and pathways in the pathophysiology of schizophrenia [5], [51]–[52]. In future work, the miRNA-target genes need to be validated in our samples by evaluating the correlation between the expression levels of miRNAs and its target genes using qRT-PCR method, and the miRNA-target interactions need to be confirmed using exogenous (e.g., to over-express certain miRNAs in neural cell lines and then examine the protein expression level of the target genes) or endogenous (e.g., conducting the manipulation of miRNAs in physiological conditions) miRNA experiments [50].

It is intriguing to note that two of the seven miRNAs, miR-34a and miR-449a, share the same seed sequence and the direction and magnitude of their fold change were similar in our study. Furthermore, they also shared similar miRNA-target genes as predicted using MAMI (**Table S5**). Among them, some have been confirmed in previous studies, such as delta-like 1 (DLL1) and jagged 1 (JAG1) genes, which are involved in NOTCH signaling pathway [53], B-cell CLL/lymphoma 2 (BCL1), which impacts outcomes after sever traumatic brain injury [54], and mitogen-activated protein kinase kinase 1 (MAP2K1), which is aberrantly expressed in the brain of suicide subjects [55]. Another intriguing finding is that despite the close alignment of miR-449b to miR-449a, the former was not selected as a predictive biomarker in this study. Previous studies indicated that the expression levels of miR-449a exceed those of miR-449b in all the systems analyzed [56]. Consistent with this, 70% of the 60 subjects of the learning set in this study had higher expression levels of the miR-449a than miRNA-449b in mononuclear leukocytes, which might explain the lack of association of miR-449b expression level with schizophrenia in this study.

Our results should be interpreted with several limitations in mind. First, since all the schizophrenia patients were receiving combinations of medications, the influences of psychopharmacologic medications on miRNA expression levels were not accounted for in this study. Second, despite our matching in age and gender, there were substantial differences between the patients and controls in educational level. Nevertheless, it is not known whether educational level has much influence on the expression levels of miRNA. Finally, subgroup analysis based on patients' endophenotypic characteristics was not conducted in this study due to limited sample size.

Our findings demonstrate the potential utility of a mononuclear leukocyte-based miRNA signature as biomarkers for schizophrenia, which could help elucidate the etiology of schizophrenia as well as proffer better treatments. The results of correlation between clinical symptoms and miRNA expression levels suggest that lymphocyte could reflect the metabolism of brain cells, and may be exploited as a neural and possible genetic probe in studies of psychiatric disorders [15]–[16]. Since a specific miRNA might be involved in coordinated regulation of protein expression in functional networks [24], this kind of biomarkers might contribute to new development of target therapeutic intervention in near future [8]. We conclude that genome-wide miRNA profiling is a feasible way to identify potential biomarkers for schizophrenia, and the seven-miRNA signature warrants further investigation.

## Materials and Methods

### Participants

From December 2007 to September 2009, we enrolled 90 patients who met the Diagnostic and Statistical Manual of Mental Disorders, Fourth Edition [57] criteria for schizophrenia at National Taiwan University Hospital, along with 60 unrelated healthy controls with a frequency matching in age and sex from a pool of staff, graduate students, and community volunteers. The sample was randomly separated into a learning set (30 cases and 30 controls) and a testing set (60 cases and 30 controls). Individuals with severe neurological abnormalities, emotional disorders, mental retardation, or prominent substance abuse problems were excluded. Each participant provided written informed consent after being given a complete description of the study. The study was approved by the institutional review board of National Taiwan University Hospital.

### Clinical assessments

All the patients were interviewed using the Chinese adaptation [34] of the Diagnostic Interview for Genetic Studies (DIGS) [58] by well-trained research assistants with background in psychology or nursing. Best estimate psychiatric diagnosis was determined independently by two psychiatrists using all available information, including the DIGS, hospital records, and the interviewer's notes. If both disagreed about a diagnosis, a third one was sought and a consensus in diagnosis was reached after discussion [59].

Each patient's clinical symptoms were rated by a research psychiatrist using the PANSS [33]. The translation of the PANSS into Chinese was described elsewhere [60] and the instrument was shown to have good inter-rater reliabilities (intraclass correlation ranging from 0.64 to 0.96) in a subsequent study [61].

### Neurocognitive assessments

Each participant's sustained attention was assessed using a CPT machine from Sunrise Systems, v.2.20 (Pembroke, MA, USA), with the procedure and reliability data being described in more detail elsewhere [34]–[35]. Briefly, numbers from 0 to 9 were randomly presented for 50 msec each, at a rate of one per second. Each subject undertook two CPT sessions: the undegraded 1–9 tasks and 25% degraded 1–9 tasks. Two signal-detection indexes of performance on the test, sensitivity (d′) and response criterion (ln β), were derived from the hit rate and false-alarm rate. Sensitivity measures an individual's ability to discriminate target stimuli from nontarget stimuli, whereas response criterion assesses the amount of perceptual evidence an individual requires prior to making a decision to respond to a stimulus as a signal.

Then each participant's executive function was assessed using a computerized version of the WCST [36]. Subjects were required to match response cards to the four stimulus cards along one of three dimensions (color, form, or number) by pressing one of the 1 to 4 number keys on the computer keyboard. Subjects were not informed of the correct sorting principle, nor were they told when the principle would shift during the test, but they were given feedback on the screen after each trial. Eight performance indices were used for subsequent analyses: Total Errors, Nonperseverative Errors, Perseverative Errors, Perseverative Responses, Categories Achieved, Trials to Complete First Category, Conceptual Level Response, and Failure to Maintain Set [62].

### Electrophysiological assessments

Each participant was assessed for auditory ERPs using P50 suppression [37] and MMN [38]. Participants were in a sound-attenuating, electrically shielded booth, and did not smoke for at least 60 min before the testing. They were seated in a comfortable recliner and instructed to relax with their eyes open and to focus on a fixation point (P50 session) or the video monitor (MMN session). The stimuli were generated and data were recorded using Neuroscan (Neuroscan, El Paso, Texas) STIM and ACQUIRE system. Electrodes were used at up to 40 recording sites (according to International 10–20 system) utilizing an electrode cap. Auditory stimuli were presented to subjects binaurally via foam insert earphones. Electroencephalography and stimulus markers were recorded continuously, while subjects were instructed to minimize eye movements and muscle artifact during the recording.

### RNA extraction and miRNA quantification

Approximately 10 ml of whole blood were collected and processed within 3 hours. Mononuclear leukocytes were separated via centrifuging with Ficoll-Paque PLUS (GE Healthcare). Since lymphocytes, monocytes, and platelets were not dense enough to penetrate into the Ficoll-Paque PLUS layer, this resulted in a concentrated band at the interface between the original blood sample and the Ficoll-Paque PLUS. This banding enabled the lymphocytes to be recovered with high yield in a small volume with little mixing with the Ficoll-Paque PLUS medium. Subsequent washing and centrifugation of the harvested cells removed platelets, plasma, and any contaminating Ficoll-Paque PLUS. The resulting cell suspension then contained highly purified, viable mononuclear leukocytes (95%±5%), in which the majority were lymphocytes (60%±20%). The suspension of mononuclear leukocytes might contain microviscles. However, the amount might be negligible since the majority of the peripheral blood microvesicles were platelet-derived [63], which were presumably washed out by the washing and centrifugation step.

Then the total RNA of these mononuclear leukocytes was extracted using TRIzol reagent (Invitrogen). The qualities of RNA extracts were found to be good, i.e., RNA integrity number (RIN)>8. In the learning set, the expression of 365 human miRNAs and endogenous control RNU48 small nuclear RNA were assayed using Multiplex RT and TaqMan® Low Density Array (TLDA) Human MicroRNA Panel v1.0 and 7900 real-time RT PCR System (Applied Biosystems). A total of 800 ng RNAs were used for each array.

The corresponding cDNAs were made using TaqMan MicroRNA reverse transcription (RT) reagents and specific primers for the miRNAs (Applied Biosystems). The amount of RNAs used for RT was 2 ng. qRT-PCR of miRNAs was performed using Taqman MicroRNA assays and 7900 real-time RT-PCR system (Applied Biosystems) with RNU48 as an endogenous control. A total of 0.8 ng RT products were used for qRT-PCR. All assays were performed in duplicate.

To confirm the comparability of TLDA-based genome-wide profiling with individual quantifications, we randomly chose 10 patients and 10 controls from the learning set and further quantified these subjects' expression levels of five miRNAs (has-miR 34a, miR-432, miR-548d, miR-659 and miR-185) using quantitative RT-PCR. Among the miRNAs detectable using TLDA, there was a significant correlation (*r* = 0.83, *P*<0.0001) in the expression levels of miRNA between the two methods (**Figure S3**).

### Statistical analyses

The expression level of each miRNA was quantified by its normalized threshold cycle number ΔC_t_, in which ΔC_t_ = [C_t_ (miRNA)]−[C_t_ (U48)], and the relative expression level was calculated as 2^−(ΔCt)^, which is commonly used in genomewide profiling studies of miRNAs [64]. Only those miRNAs detectable in the peripheral mononuclear leukocytes of ≥30% of the samples of either the schizophrenia patients or the controls were subsequently analyzed. To select differentially expressed miRNAs for further classification, a two-sided Wilcoxon rank-sum test was used to compare the two groups' expression levels with a threshold *P* value of 0.05. Stepwise logistic regression analyses were then used to select a miRNA signature with the maximum acceptable α limit for adding a variable being 0.1 and the minimum acceptable α limit for removing a variable being 0.15.

We used two different methods in a supervised classification with leave-one-out cross-validation [65] to assess the prediction accuracy of the miRNA-signature in the learning set: logistic regression and SVM. Logistic regression allows for non-linear discriminant analysis and is more robust against departures from normality than discriminant analysis, whereas SVM is particularly useful for high-dimensional classification problems [32]. A supervised classification with leave-one-out cross-validation can provide a nearly unbiased estimate of the true error rate [65]. In these analyses, a confounder score method [66] was used to adjust for age, gender, education, and tobacco smoking. To assess the robustness of the predictive utility of the seven-miRNA signature, two kinds of permutations were conducted. First, keeping the seven-miRNA signature, we randomly permuted the disease status among the learning set and the testing set, respectively, for 10,000 times and the number of the permuted AUC exceeding the observed AUC was counted as permuted significance level. Second, keeping the disease status of the learning set of subjects, a certain number of miRNAs, ranging from 3 to 9, were randomly selected from the pool of 221 miRNAs and the corresponding AUC for the model without and with control of potential confounders, respectively, were calculated.

To help visualize the differential expression pattern, we adopted the method of matrix visualization using the software program Generalized Association Plots [67] to compare the miRNA expression levels between cases and controls.

For correlation analysis, either a Pearson (between array-based expression level and qRT-PCR expression level) or a Spearman (between the expression levels of miRNAs and scores on the clinical symptoms, neurocognitive tests, and ERPs) correlation coefficient was used. The expression level of individual miRNA was standardized by subtraction from the group mean followed by division by the standard deviation within each platform. Then the standardized expression levels of miRNAs from two platforms were pooled.

### Target genes prediction and functional profiling

The potential target genes regulated by the miRNAs were predicted using two different methods: the MAMI MicroRNA Meta-Predictor [39] and TargetCombo [40]. These two methods incorporate several popular prediction algorithms including miRs, Targets, microT [68], PicTar [69], TargetScanS [70], miRTarget [71], and miRanda [72]. For the predicted target genes, their functions and putative pathways were explored using the IPA version 8 (Ingenuity® Systems). Fisher's exact test was used to calculate the significance of the association between the data set and the biological function or canonical pathway. The false discovery rate procedure were used to control for the multiple hypothesis testing [73].

## Supporting Information

Figure S1 The histogram of the AUCs in 100,000 permutations of random selection of seven miRNAs from the pool of 221 miRNAs for (a) the model without adjustment and (b) the model with adjustment for confounders (age, gender, education, and tobacco smoking).
(TIF)

Figure S2 The canonical pathways revealed by means of using the software Ingenuity Pathways Analysis to be significantly (*P*<0.005) associated with the 619 miRNA-target genes predicted using MAMI. The pathways that remained significantly associated with the predicted target genes after corrections for multiple testing using the false discovery rate (p<0.001) were marked by an asterisk.
(TIF)

Figure S3 The relations in the five miRNAs (has-miR 34a, miR-432, miR-548d, miR-659 and miR-185) expression levels between two platforms of miRNA quantification, the array-based TLDA vs. individual quantification using quantitative RT-PCR in the learning set of 10 schizophrenia patients and 10 controls. The Pearson correlation in detectable miRNA expression levels between the two methods was *r* = 0.83 (*P*<0.0001).
(TIF)

Table S1 Demographic characteristics of schizophrenia patients and healthy controls in the learning set and testing set, respectively.
(DOC)

Table S2 Summary statistics of the area under the curve (AUC) of receiver operating characteristics of 100,000 permutations of random selection of a certain number of miRNAs from the pool of 221 miRNAs in the learning set of 30 schizophrenia patients and 30 controls.
(DOC)

Table S3 Measures on clinical, neurocognitive, and auditory event related potentials in schizophrenia patients and normal controls.
(DOC)

Table S4 Summary of miRNA-target prediction using two different methods: the MAMI MicroRNA Meta-Predictor and TargetCombo.
(DOC)

Table S5 The common miRNA-target genes that were predicted by MAMI MicroRNA Meta-Predictor shared by different miRNAs.
(DOC)
