# Supplementary figures and images for: MicroRNA Expression Aberration as Potential Peripheral Blood Biomarkers for Schizophrenia
Source: PLoS One. 2011 Jun 29;6(6):e21635. doi: 10.1371/journal.pone.0021635 (PMC3126851; doi:10.1371/journal.pone.0021635)

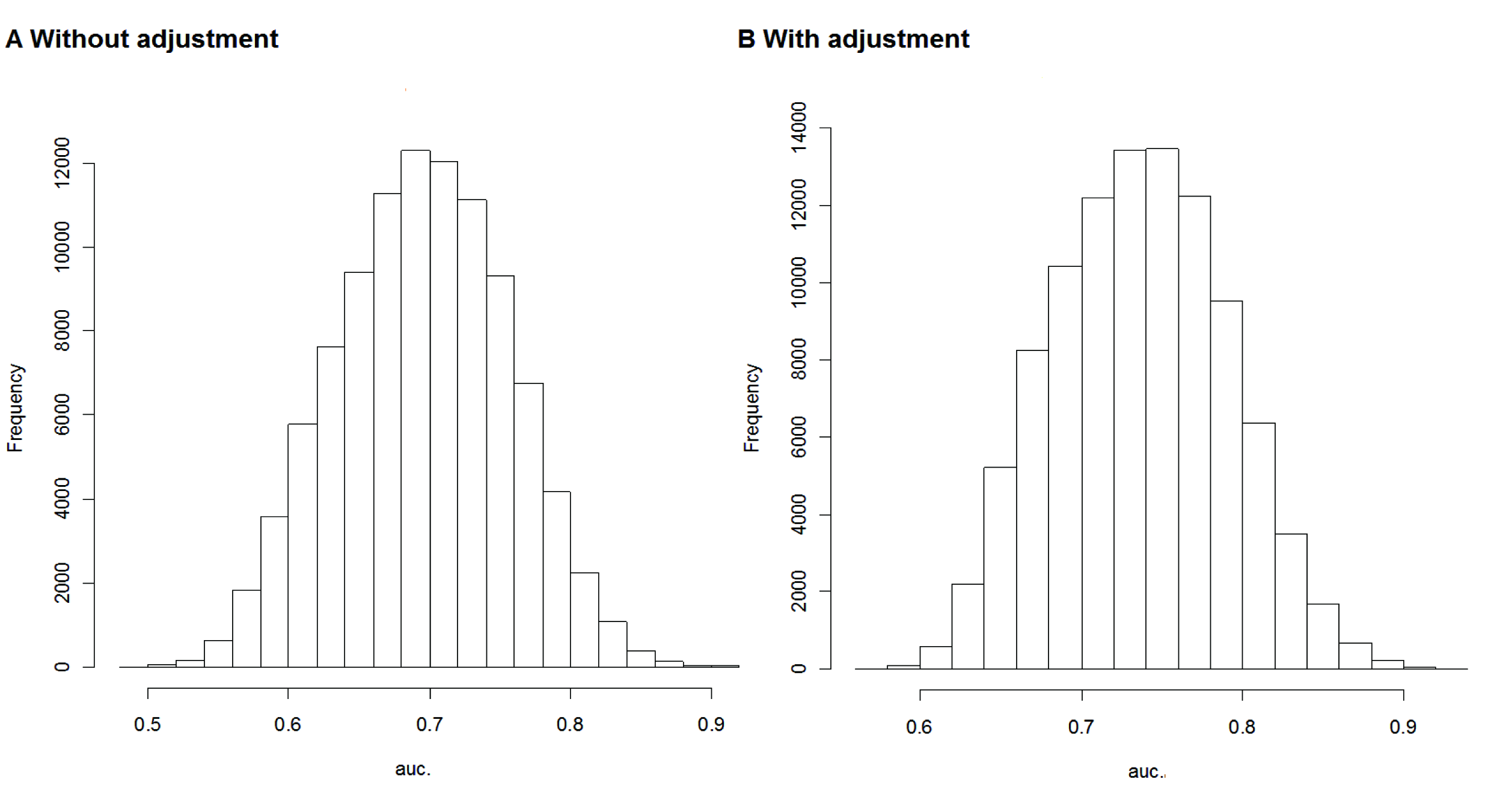

Supplement: Figure S1 — The histogram of the AUCs in 100,000 permutations of random selection of seven miRNAs from the pool of 221 miRNAs for (a) the model without adjustment and (b) the model with adjustment for confounders (age, gender, education, and tobacco smoking). (TIF) [file pone.0021635.s001.tif]

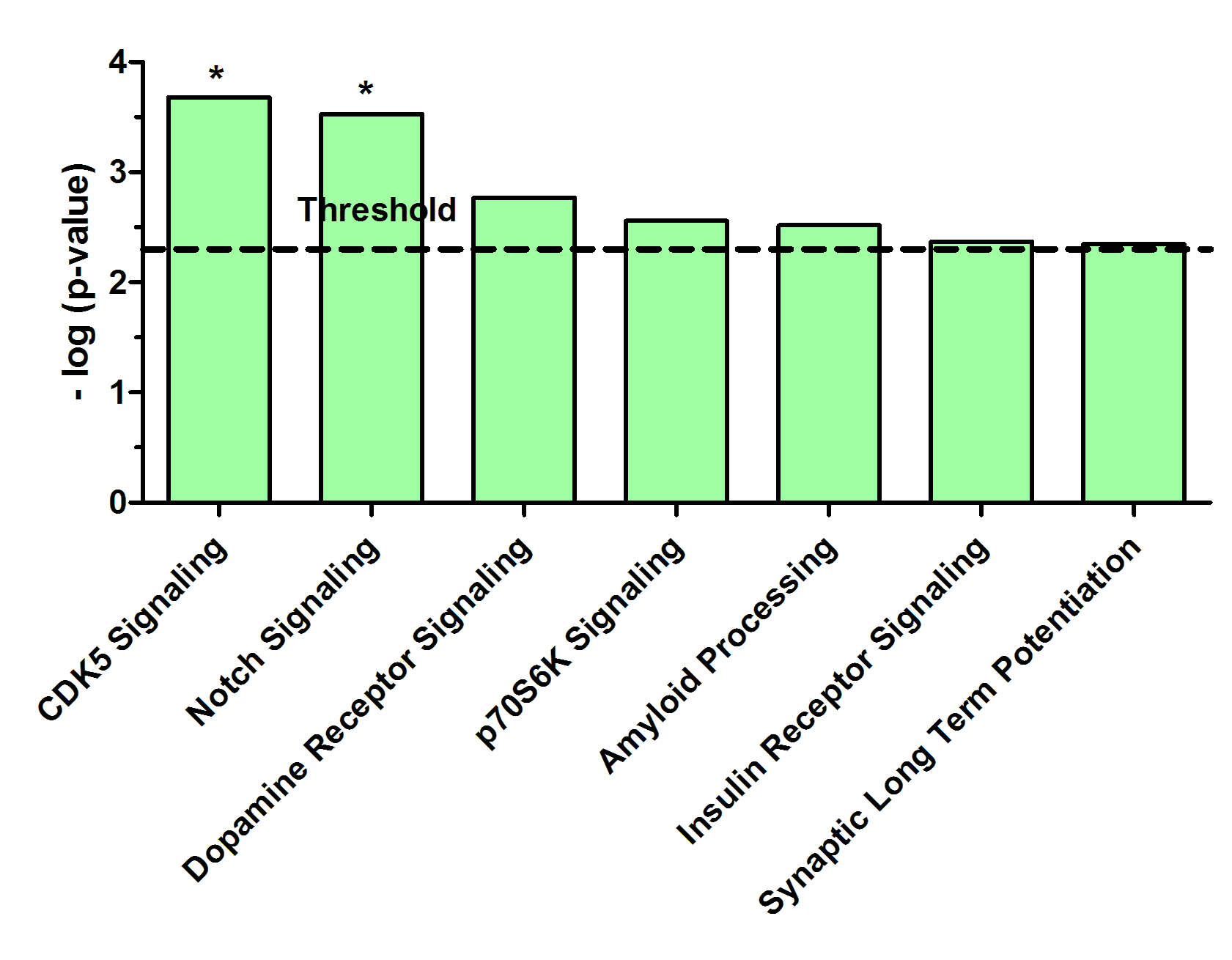

Supplement: Figure S2 — The canonical pathways revealed by means of using the software Ingenuity Pathways Analysis to be significantly (P<0.005) associated with the 619 miRNA-target genes predicted using MAMI. The pathways that remained significantly associated with the predicted target genes after corrections for multiple testing using the false discovery rate (p<0.001) were marked by an asterisk. (TIF) [file pone.0021635.s002.tif]

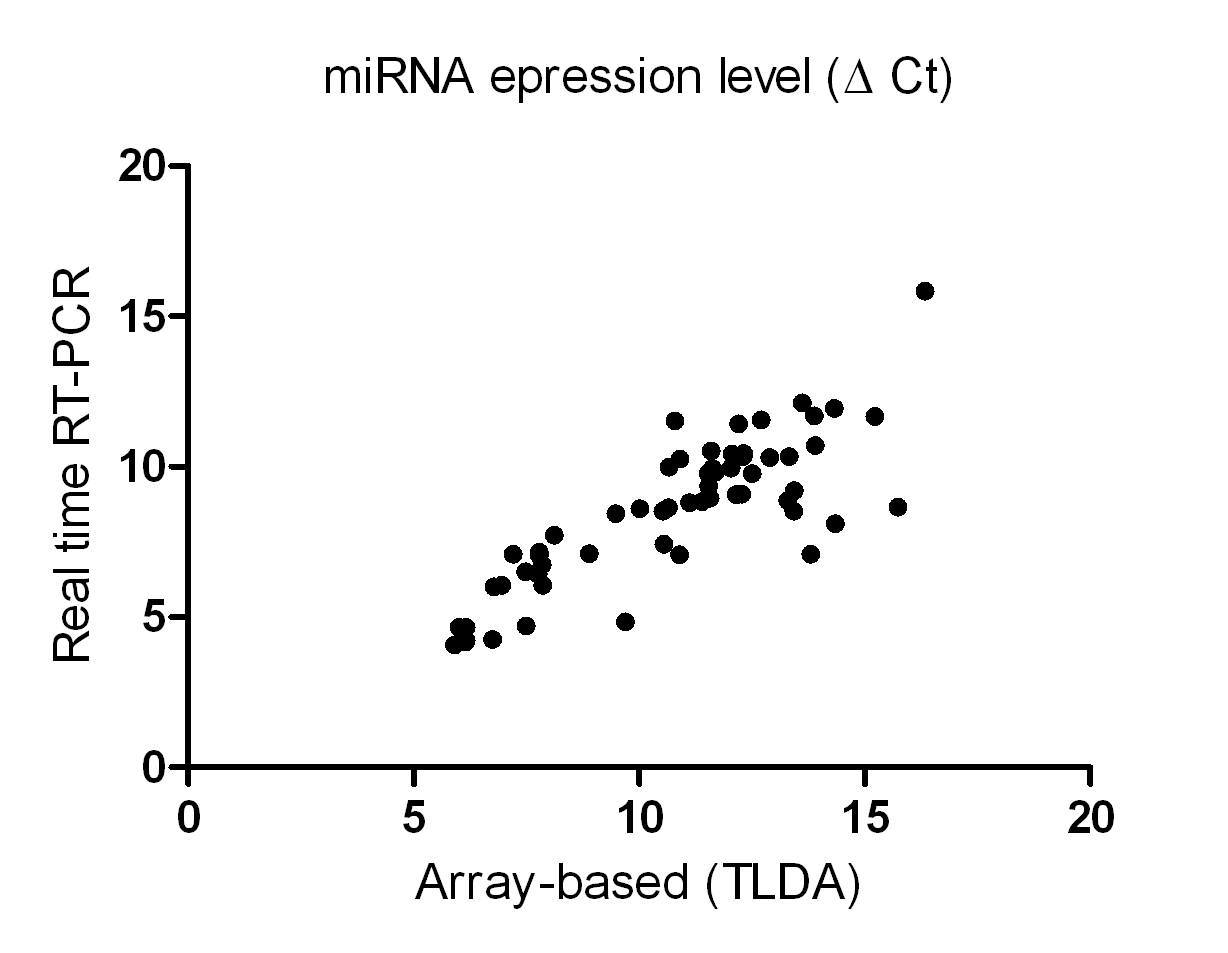

Supplement: Figure S3 — The relations in the five miRNAs (has-miR 34a, miR-432, miR-548d, miR-659 and miR-185) expression levels between two platforms of miRNA quantification, the array-based TLDA vs. individual quantification using quantitative RT-PCR in the learning set of 10 schizophrenia patients and 10 controls. The Pearson correlation in detectable miRNA expression levels between the two methods was r = 0.83 (P<0.0001). (TIF) [file pone.0021635.s003.tif]
