# Supplementary material for: MicroRNA Expression Aberration as Potential Peripheral Blood Biomarkers for Schizophrenia
Source: PLoS One. 2011 Jun 29;6(6):e21635. doi: 10.1371/journal.pone.0021635 (PMC3126851; doi:10.1371/journal.pone.0021635)
Supplement: Table S1 — Demographic characteristics of schizophrenia patients and healthy controls in the learning set and testing set, respectively. (DOC) [file pone.0021635.s004.doc]

**Table S1.** Demographic characteristics of schizophrenia patients and healthy controls in the learning set and testing set, respectively.

|  | Learning set | | | |  | Testing set | | | |
| --- | --- | --- | --- | --- | --- | --- | --- | --- | --- |
|  | Cases (n = 30) | | Controls (n = 30) | |  | Cases (n = 60) | | Controls (n = 30) | |
| Variable | N (%) | | N (%) | |  | N (%) | | N (%) | |
| Gender |  |  |  |  |  |  |  |  |  |
| Female | 18 | (60) | 18 | (60) |  | 32 | (53.3) | 17 | (56.7) |
| Male | 12 | (40) | 12 | (40) |  | 28 | (46.7) | 13 | (43.3) |
| Age |  |  |  |  |  |  |  |  |  |
| 20-25 | 2 | (6.7) | 4 | (13.3) |  | 2 | (5) | 2 | (6.7) |
| 25-35 | 14 | (46.7) | 12 | (40) |  | 22 | (36.7) | 12 | (40) |
| 35-45 | 5 | (16.7) | 4 | (13.3) |  | 21 | (35) | 7 | (23.3) |
| 45-55 | 7 | (23.3) | 8 | (26.7) |  | 12 | (20) | 7 | (23.3) |
| 55-65 | 2 | (6.7) | 2 | (6.7) |  | 2 | (3.3) | 2 | (6.7) |
| Education (years)* |  |  |  |  |  |  |  |  |  |
| ≤ 12 | 10 | (33.3) | 9 | (30) |  | 29 | (48.3) | 0 | (0) |
| 13-16 | 17 | (56.7) | 5 | (16.7) |  | 24 | (40) | 26 | (86.7) |
| ≥ 17 | 3 | (10) | 16 | (53.3) |  | 7 | (11.7) | 4 | (13.3) |
| Smoking |  |  |  |  |  |  |  |  |  |
| No | 25 | (86.2) | 28 | (93.3) |  | 44 | (78.6)* | 28 | (96.6)* |
| Yes | 4 | (13.8) | 2 | (6.7) |  | 12 | (21.4)* | 1 | (3.4)* |
| Age of onset, mean (SD) | 23.7 | (7.9) | − |  |  | 24.6 | (7.6) | − |  |
| * p <0.05 for χ^2^ test or Fisher exact test comparing cases with controls | | | | | | | | |  |
