# Supplementary material for: MicroRNA Expression Aberration as Potential Peripheral Blood Biomarkers for Schizophrenia
Source: PLoS One. 2011 Jun 29;6(6):e21635. doi: 10.1371/journal.pone.0021635 (PMC3126851; doi:10.1371/journal.pone.0021635)
Supplement: Table S2 — Summary statistics of the area under the curve (AUC) of receiver operating characteristics of 100,000 permutations of random selection of a certain number of miRNAs from the pool of 221 miRNAs in the learning set of 30 schizophrenia patients and 30 controls. (DOC) [file pone.0021635.s005.doc]

**Table S2.** Summary statistics of the area under the curve (AUC) of receiver operating characteristics of 100,000 permutations of random selection of a certain number of miRNAs from the pool of 221 miRNAs in the learning set of 30 schizophrenia patients and 30 controls.

| No. of | AUC of Unadjusted Model | | | AUC of Adjusted Model^a^ | | |
| --- | --- | --- | --- | --- | --- | --- |
| miRNAs | P-value^b^ | Mean | SD | P-value^b^ | Mean | SD |
| 9 | 0.00006 | 0.73 | 0.059 | 0.00038 | 0.77 | 0.054 |
| 8 | 0.00001 | 0.71 | 0.06 | 0.00017 | 0.754 | 0.054 |
| 7 | 0 | 0.69 | 0.06 | 0.00001 | 0.737 | 0.05 |
| 6 | 0 | 0.68 | 0.06 | 0.00001 | 0.72 | 0.05 |
| 5 | 0 | 0.66 | 0.06 | 0 | 0.70 | 0.05 |
| 4 | 0 | 0.63 | 0.06 | 0 | 0.68 | 0.049 |
| 3 | 0 | 0.60 | 0.05 | 0 | 0.66 | 0.45 |

^a^A confounder score method was used to adjust for age, gender, education, and tobacco smoking in the logistic regression analysis.

^b^Counted as the number of permuted AUC > the observed AUC (0.93 for both unadjusted and adjusted model)
