# Supplementary material for: MicroRNA Expression Aberration as Potential Peripheral Blood Biomarkers for Schizophrenia
Source: PLoS One. 2011 Jun 29;6(6):e21635. doi: 10.1371/journal.pone.0021635 (PMC3126851; doi:10.1371/journal.pone.0021635)
Supplement: Table S3 — Measures on clinical, neurocognitive, and auditory event related potentials in schizophrenia patients and normal controls. (DOC) [file pone.0021635.s006.doc]

**Table S3.** Measures on clinical, neurocognitive, and auditory event related potentials in schizophrenia patients and normal controls.

|  | Controls | |  | Cases | |  | Comparison^b^ |
| --- | --- | --- | --- | --- | --- | --- | --- |
| Variable | Mean | (SD) |  | Mean | (SD) |  | P value |
| Positive and Negative Syndrome Scale | n = 55 | |  | n = 83 | |  |  |
| Positive scale | − | − |  | 11.86 | (3.92) |  | − |
| Negative scale | − | − |  | 14.75 | (6.43) |  | − |
| Undegraded CPT^a^ | n = 50 | |  | n = 78 | |  |  |
| Hit rate | 0.01 | (0.78) |  | −0.54 | (1.49) |  | 0.03 |
| False alarm rate | 0.58 | (4.25) |  | 0.67 | (2.07) |  | ns^c^ |
| d' | 0.05 | (1.03) |  | −0.69 | (1.28) |  | 0.0002 |
| ln β | −0.18 | (0.87) |  | −0.24 | (1.50) |  | ns |
| Reaction time (centi-sec) | 0.16 | (1.1) |  | 0.25 | (1.04) |  | ns |
| Degraded CPT^a^ | n = 50 | |  | n = 77 | |  |  |
| Hit rate | −0.14 | (0.87) |  | −0.67 | (1.30) |  | 0.04 |
| False alarm rate | 0.11 | (0.53) |  | 0.70 | (1.40) |  | 0.01 |
| d' | −0.08 | (1.03) |  | −0.84 | (1.21) |  | 0.0005 |
| ln β | −0.26 | (0.73) |  | −0.46 | (1.15) |  | ns |
| Reaction time (centi-sec) | 0.27 | (0.9) |  | 0.41 | (1.1) |  | ns |
| Wisconsin Card Sorting Test^a^ | n = 55 | |  | n = 78 | |  |  |
| Total errors | −0.38 | (0.97) |  | 0.64 | (1.15) |  | <.0001 |
| Perseverative responses | −0.15 | (1.02) |  | 0.75 | (1.41) |  | <.0001 |
| Non-perseverative errors | −0.37 | (0.79) |  | 0.05 | (0.96) |  | 0.007 |
| Perseverative errors | −0.17 | (1.02) |  | 0.76 | (1.39) |  | <.0001 |
| Categories achieved | 0.46 | (0.94) |  | −0.57 | (1.05) |  | <.0001 |
| Trials to complete first category | −0.23 | (0.40) |  | −0.01 | (0.74) |  | ns |
| Conceptual level response | 0.38 | (0.98) |  | −0.63 | (1.14) |  | <.0001 |
| Failure to maintain set | −0.17 | (0.85) |  | −0.06 | (0.92) |  | ns |
| Learning to learn | 0.46 | (0.94) |  | -0.57 | (1.05) |  | ns |
| Mismatch negativity | n = 55 | |  | n = 70 | |  |  |
| Cz | −0.93 | (0.96) |  | −0.38 | (0.79) |  | 0.002 |
| Fz | −0.92 | (0.91) |  | −0.45 | (0.65) |  | 0.001 |
| FCz | −1.04 | (1.03) |  | −0.42 | (0.77) |  | 0.0005 |
| P50 suppression | n = 56 | |  | n = 69 | |  |  |
| Gating ratio | 0.43 | (0.46) |  | 0.43 | (0.39) |  | ns |
| Gating difference | 1.45 | (1.12) |  | 1. 45 | (1.14) |  | ns |

^a^ In adjusted z scores, which were derived by means of standardizing the raw scores with adjustments for sex, age, and education against a community sample of 345 individuals for the CPT indices and another group of 392 healthy individuals for the WCST indices.

^b^ Wilcoxon rank sum test

^c^ ns = non-significant
