# Supplementary material for: MicroRNA Expression Aberration as Potential Peripheral Blood Biomarkers for Schizophrenia
Source: PLoS One. 2011 Jun 29;6(6):e21635. doi: 10.1371/journal.pone.0021635 (PMC3126851; doi:10.1371/journal.pone.0021635)
Supplement: Table S4 — Summary of miRNA-target prediction using two different methods: the MAMI MicroRNA Meta-Predictor and TargetCombo. (DOC) [file pone.0021635.s007.doc]

**Table S4.** Summary of miRNA-target prediction using two different methods: the MAMI MicroRNA Meta-Predictor and TargetCombo

|  | Numbers of target genes | |
| --- | --- | --- |
|  | MAMI | TargetCombo |
| **Setting**^a^ | Sensitivity = 0.82, Specificity = 0.4,  Percentile = 22 | Target Type: Union (DIANA-microT, PicTar, TargetScanS, miRanda) |
| **miRNAs that have predicted miRNA-target genes** | | |
| hsa-miR-34a | 496 | 999 |
| hsa-miR-449a | 220 | 15 |
| has-miR-432 | 35 | 0 |
| Total target genes | 751 | 1014 |
| **Mapped to IPA version 8** | 748 | 849 |
| Common target genes |  |  |
| hsa-miR-34a and hsa-miR-449 | 128 | 330 |
| hsa-miR-34a and hsa-miR-432 | 1 | 0 |
| has-miR-432 and hsa-miR-449 | 1 | 0 |
| Total unique target genes | 621 | 684 |
| **Mapped to IPA version 8** | 619 | 520 |

^a^Default was used for each method
