# Supplementary material for: MicroRNA Expression Aberration as Potential Peripheral Blood Biomarkers for Schizophrenia
Source: PLoS One. 2011 Jun 29;6(6):e21635. doi: 10.1371/journal.pone.0021635 (PMC3126851; doi:10.1371/journal.pone.0021635)
Supplement: Table S5 — The common miRNA-target genes that were predicted by MAMI MicroRNA Meta-Predictor shared by different miRNAs. (DOC) [file pone.0021635.s008.doc]

**Table S5.** The common miRNA-target genes that were predicted by MAMI MicroRNA Meta-Predictor shared by different miRNAs

| miRNAs | Symbol | Entrez Gene Name | Location | | Type(s) |
| --- | --- | --- | --- | --- | --- |
| miR34a,miR449 (n = 128) | | | | | |
|  | ACBD3 | acyl-CoA binding domain containing 3 | | Cytoplasm | other |
|  | ACTR1A | ARP1 actin-related protein 1 homolog A, centractin alpha (yeast) | | Cytoplasm | other |
|  | AHSG | alpha-2-HS-glycoprotein | | Extracellular Space | other |
|  | AIP | aryl hydrocarbon receptor interacting protein | | Nucleus | transcription regulator |
|  | AKAP6 | A kinase (PRKA) anchor protein 6 | | Nucleus | other |
|  | AP1S2 | adaptor-related protein complex 1, sigma 2 subunit | | Cytoplasm | transporter |
|  | ATP6V1A | ATPase, H+ transporting, lysosomal 70kDa, V1 subunit A | | Cytoplasm | transporter |
|  | BAALC | brain and acute leukemia, cytoplasmic | | Cytoplasm | other |
|  | C2 | complement component 2 | | Extracellular Space | peptidase |
|  | CCDC92 | coiled-coil domain containing 92 | | Cytoplasm | enzyme |
|  | CCL13 | chemokine (C-C motif) ligand 13 | | Extracellular Space | cytokine |
|  | CDH3 | cadherin 3, type 1, P-cadherin (placental) | | Plasma Membrane | other |
|  | CIAO1 | cytosolic iron-sulfur protein assembly 1 | | Nucleus | transcription regulator |
|  | CSF1R | colony stimulating factor 1 receptor | | Plasma Membrane | kinase |
|  | DDX17 | DEAD (Asp-Glu-Ala-Asp) box polypeptide 17 | | Nucleus | enzyme |
|  | DGKZ | diacylglycerol kinase, zeta | | Cytoplasm | kinase |
|  | DLL1 | delta-like 1 (Drosophila) | | Plasma Membrane | enzyme |
|  | DPP3 | dipeptidyl-peptidase 3 | | Cytoplasm | peptidase |
|  | DPYSL4 | dihydropyrimidinase-like 4 | | Cytoplasm | enzyme |
|  | EFNB1 | ephrin-B1 | | Plasma Membrane | other |
|  | EI24 | etoposide induced 2.4 mRNA | | unknown | other |
|  | EME1 | essential meiotic endonuclease 1 homolog 1 (S. pombe) | | Nucleus | other |
|  | EML5 | echinoderm microtubule associated protein like 5 | | unknown | other |
|  | FAM38A | family with sequence similarity 38, member A | | unknown | other |
|  | FAM76A | family with sequence similarity 76, member A | | unknown | other |
|  | FKBP8 | FK506 binding protein 8, 38kDa | | Cytoplasm | other |
|  | FOXG1 | forkhead box G1 | | Nucleus | transcription regulator |
|  | FOXP1 | forkhead box P1 | | Nucleus | transcription regulator |
|  | FUT8 | fucosyltransferase 8 (alpha (1,6) fucosyltransferase) | | Cytoplasm | enzyme |
|  | GFER | growth factor, augmenter of liver regeneration | | Nucleus | other |
|  | GNB1 | guanine nucleotide binding protein (G protein), beta polypeptide 1 | | Plasma Membrane | enzyme |
|  | GPR98 | G protein-coupled receptor 98 | | Plasma Membrane | G-protein coupled receptor |
|  | GPS2 | G protein pathway suppressor 2 | | Nucleus | other |
|  | GPX5 | glutathione peroxidase 5 (epididymal androgen-related protein) | | Extracellular Space | enzyme |
|  | GREM2 | gremlin 2 | | Extracellular Space | other |
|  | GRK6 | G protein-coupled receptor kinase 6 | | Plasma Membrane | kinase |
|  | HAAO | 3-hydroxyanthranilate 3,4-dioxygenase | | Cytoplasm | enzyme |
|  | HIST4H4 | histone cluster 1, H4c | | Nucleus | other |
|  | HSPB6 | heat shock protein, alpha-crystallin-related, B6 | | Cytoplasm | other |
|  | IFNB1 | interferon, beta 1, fibroblast | | Extracellular Space | cytokine |
|  | IFNW1 | interferon, omega 1 | | Extracellular Space | cytokine |
|  | IGHM | immunoglobulin heavy constant mu | | Plasma Membrane | transmembrane receptor |
|  | Igkv1-117 | immunoglobulin kappa chain variable 1-117 | | unknown | other |
|  | INF2 | inverted formin, FH2 and WH2 domain containing | | Cytoplasm | other |
|  | JAG1 | jagged 1 | | Extracellular Space | growth factor |
|  | JAKMIP1 | janus kinase and microtubule interacting protein 1 | | Cytoplasm | other |
|  | JOSD2 | Josephin domain containing 2 | | unknown | other |
|  | LDHA | lactate dehydrogenase A | | Cytoplasm | enzyme |
|  | LIMD2 | LIM domain containing 2 | | unknown | other |
|  | LMNA | lamin A/C | | Nucleus | other |
|  | MAP2K1 | mitogen-activated protein kinase kinase 1 | | Cytoplasm | kinase |
|  | MARCKSL1 | MARCKS-like 1 | | Cytoplasm | other |
|  | MBLAC1 | metallo-beta-lactamase domain containing 1 | | unknown | other |
|  | MOV10L1 | Mov10l1, Moloney leukemia virus 10-like 1, homolog (mouse) | | Nucleus | enzyme |
|  | MPP2 | membrane protein, palmitoylated 2 (MAGUK p55 subfamily member 2) | | Plasma Membrane | kinase |
|  | MTA2 | metastasis associated 1 family, member 2 | | Nucleus | transcription regulator |
|  | MUC5AC/MUC5B | mucin 5AC, oligomeric mucus/gel-forming | | Extracellular Space | peptidase |
|  | Mucl1/Spt1 | salivary protein 1 | | Plasma Membrane | other |
|  | MYL9 | myosin, light chain 9, regulatory | | Cytoplasm | other |
|  | MYO18A | myosin XVIIIA | | Cytoplasm | other |
|  | MYO1F | myosin IF | | Cytoplasm | other |
|  | NAT10 | N-acetyltransferase 10 (GCN5-related) | | Nucleus | enzyme |
|  | NCAPD3 | non-SMC condensin II complex, subunit D3 | | Nucleus | other |
|  | NCDN | neurochondrin | | Cytoplasm | other |
|  | NDUFC2 | NADH dehydrogenase (ubiquinone) 1, subcomplex unknown, 2, 14.5kDa | | Cytoplasm | enzyme |
|  | NDUFS4 | NADH dehydrogenase (ubiquinone) Fe-S protein 4, 18kDa (NADH-coenzyme Q reductase) | | Cytoplasm | enzyme |
|  | NFKBIA | nuclear factor of kappa light polypeptide gene enhancer in B-cells inhibitor, alpha | | Cytoplasm | other |
|  | NHLRC2 | NHL repeat containing 2 | | unknown | enzyme |
|  | NRIP3 | nuclear receptor interacting protein 3 | | unknown | other |
|  | NUP188 | nucleoporin 188kDa | | Nucleus | other |
|  | PACS1 | phosphofurin acidic cluster sorting protein 1 | | Cytoplasm | other |
|  | PCM1 | pericentriolar material 1 | | Cytoplasm | other |
|  | PDGFRA | platelet-derived growth factor receptor, alpha polypeptide | | Plasma Membrane | kinase |
|  | PEX16 | peroxisomal biogenesis factor 16 | | Cytoplasm | other |
|  | PGD | phosphogluconate dehydrogenase | | Cytoplasm | enzyme |
|  | PGM1 | phosphoglucomutase 1 | | Cytoplasm | enzyme |
|  | PKP4 | plakophilin 4 | | Plasma Membrane | other |
|  | PLCG1 | phospholipase C, gamma 1 | | Cytoplasm | enzyme |
|  | PLS3 | plastin 3 | | Cytoplasm | other |
|  | PMF1 | polyamine-modulated factor 1 | | Nucleus | transcription regulator |
|  | PNOC | prepronociceptin | | Extracellular Space | other |
|  | POU6F1 | POU class 6 homeobox 1 | | Nucleus | transcription regulator |
|  | PPP1R11 | protein phosphatase 1, regulatory (inhibitor) subunit 11 | | Cytoplasm | other |
|  | PPP1R12B | protein phosphatase 1, regulatory (inhibitor) subunit 12B | | Cytoplasm | phosphatase |
|  | PPP1R14D | protein phosphatase 1, regulatory (inhibitor) subunit 14D | | Cytoplasm | other |
|  | propofol |  | | unknown | chemical drug |
|  | PSMD3 | proteasome (prosome, macropain) 26S subunit, non-ATPase, 3 | | Cytoplasm | other |
|  | PTPN22 | protein tyrosine phosphatase, non-receptor type 22 (lymphoid) | | Cytoplasm | phosphatase |
|  | PTRH1 | peptidyl-tRNA hydrolase 1 homolog (S. cerevisiae) | | Cytoplasm | enzyme |
|  | RALGDS | ral guanine nucleotide dissociation stimulator | | Cytoplasm | other |
|  | RALY | RNA binding protein, autoantigenic (hnRNP-associated with lethal yellow homolog (mouse)) | | Nucleus | other |
|  | RASA4 | RAS p21 protein activator 4 | | Cytoplasm | other |
|  | RASGEF1C | RasGEF domain family, member 1C | | unknown | other |
|  | RCVRN | recoverin | | Cytoplasm | other |
|  | RDH11 | retinol dehydrogenase 11 (all-trans/9-cis/11-cis) | | Cytoplasm | enzyme |
|  | RGS17 | regulator of G-protein signaling 17 | | Cytoplasm | other |
|  | RPL13A | ribosomal protein L13a | | Cytoplasm | other |
|  | RPS6KA4 | ribosomal protein S6 kinase, 90kDa, polypeptide 4 | | Cytoplasm | kinase |
|  | SAMD3 | sterile alpha motif domain containing 3 | | unknown | other |
|  | SAR1A | SAR1 homolog A (S. cerevisiae) | | Cytoplasm | enzyme |
|  | SDR9C7 | short chain dehydrogenase/reductase family 9C, member 7 | | unknown | enzyme |
|  | SEC61A1 | Sec61 alpha 1 subunit (S. cerevisiae) | | Cytoplasm | transporter |
|  | SEMA4F | sema domain, immunoglobulin domain (Ig), transmembrane domain (TM) and short cytoplasmic domain, (semaphorin) 4F | | Plasma Membrane | other |
|  | SEPT1 | septin 1 | | Cytoplasm | enzyme |
|  | SERPINF2 | serpin peptidase inhibitor, clade F (alpha-2 antiplasmin, pigment epithelium derived factor), member 2 | | Extracellular Space | other |
|  | SGTA | small glutamine-rich tetratricopeptide repeat (TPR)-containing, alpha | | Cytoplasm | other |
|  | SHKBP1 | SH3KBP1 binding protein 1 | | unknown | other |
|  | SIRT6 | sirtuin 6 | | Nucleus | enzyme |
|  | SLC2A13 | solute carrier family 2 (facilitated glucose transporter), member 13 | | Plasma Membrane | transporter |
|  | SPEG | SPEG complex locus | | Nucleus | kinase |
|  | SPSB2 | splA/ryanodine receptor domain and SOCS box containing 2 | | unknown | other |
|  | STARD3 | StAR-related lipid transfer (START) domain containing 3 | | Cytoplasm | transporter |
|  | SV2A | synaptic vesicle glycoprotein 2A | | Cytoplasm | transporter |
|  | SYT1 | synaptotagmin I | | Cytoplasm | transporter |
|  | TAF5 | TAF5 RNA polymerase II, TATA box binding protein (TBP)-associated factor, 100kDa | | Nucleus | transcription regulator |
|  | TCF7 | transcription factor 7 (T-cell specific, HMG-box) | | Nucleus | transcription regulator |
|  | TENC1 | tensin like C1 domain containing phosphatase (tensin 2) | | Plasma Membrane | other |
|  | TGIF2 | TGFB-induced factor homeobox 2 | | Nucleus | transcription regulator |
|  | TH | tyrosine hydroxylase | | Cytoplasm | enzyme |
|  | TIMP4 | TIMP metallopeptidase inhibitor 4 | | Extracellular Space | other |
|  | TMEM22 | transmembrane protein 22 | | Cytoplasm | other |
|  | TMEM48 | transmembrane protein 48 | | Nucleus | other |
|  | TMEM55A | transmembrane protein 55A | | unknown | other |
|  | TMSB10/TMSB4X | thymosin beta 4, X-linked | | Cytoplasm | other |
|  | TRAPPC6A | trafficking protein particle complex 6A | | Cytoplasm | other |
|  | USP39 | ubiquitin specific peptidase 39 | | Nucleus | peptidase |
|  | VWA5B2 | von Willebrand factor A domain containing 5B2 | | unknown | other |
|  | XYLT1 | xylosyltransferase I | | Cytoplasm | enzyme |
| miR34a,miR432 (n = 1) | | | | | |
|  | DAB2IP | DAB2 interacting protein | Plasma Membrane | | other |
| miR449,miR432 (n = 1) | | | | | |
|  | PPP1R12B | protein phosphatase 1, regulatory (inhibitor) subunit 12B | Cytoplasm | | phosphatase |
